# Meeting Review: Epigenetics in Development and Disease

**DOI:** 10.1002/cfg.176

**Published:** 2002-06

**Authors:** Andrew J. Mungall

**Affiliations:** The Wellcome Trust Sanger Institute Wellcome Trust Genome Campus Hinxton, Cambridge CB10 1SA UK

## Abstract

The 2002 Keystone symposia held in Taos, New Mexico (21–26 February) saw the convergence of two related fields; Epigenetics in Development and Disease, and RNA Interference, Cosuppression and Related Phenomena. The meeting highlights presented here concentrate upon the sessions within the Epigenetics in Development and Disease meeting, although there were joint sessions which will also be discussed. Of course epigenetic regulation is not restricted to the vertebrates but I have chosen, rightly or wrongly, to limit the highlights to those concerning vertebrates.


**Comparative and Functional Genomics**
*Comp Funct Genom* 2002; **3**: 277–281.
Published online 9 May 2002 in Wiley InterScience (www.interscience.wiley.com). **DOI:** 10.1002/cfg.176

**Feature**

### Meeting Review: Epigenetics in Development and Disease

21–26 February 2002, Taos Convention Center, Taos, New Mexico, USA

Andrew J. Mungall*
*The Wellcome Trust Sanger Institute, Wellcome Trust Genome Campus, Hinxton, Cambridge CB10 1SA, UK*

**Correspondence to:*
*The Wellcome Trust Sanger Institute, Wellcome Trust Genome Campus, Hinxton, Cambridge CB10 1SA, UK.*
*E-mail: ajm@sanger.ac.uk*

Received: 19 April 2002
Accepted: 23 April 2002

### Abstract

**The 2002 Keystone symposia held in Taos, New Mexico (21–26 February) saw the convergence of two related fields; Epigenetics in Development and Disease, and RNA Interference, Cosuppression and Related Phenomena. The meeting highlights presented here concentrate upon the sessions within the Epigenetics in Development and Disease meeting, although there were joint sessions which will also be discussed. Of course epigenetic regulation is not restricted to the vertebrates but I have chosen, rightly or wrongly, to limit the highlights to those concerning vertebrates. Copyright © 2002 John Wiley & Sons, Ltd.**

**Keywords:** **epigenetics; Meeting Review; imprinting**

Sadly the principle organiser of the meeting, **Alan Wolffe**, died in 2001. Internationally recognised for his research on chromatin structure and its role in the regulation of gene expression, Dr Wolffe was paid many tributes from friends and colleagues throughout the meeting.

As a newcomer to the field of Epigenetics, having spent nine years working in the mapping and sequencing of the human genome and its four bases Adenine (A), Cytosine (C), Guanine (G) and Thymine (T) it was clear that I'd been missing the all important $5^{th}$ base of DNA, namely 5-methylcytosine ($m^5C$) and the epigenome. The importance of $m^5C$ in both development and disease would become very clear in the days to come.

In his keynote address, **Peter Jones** (**University of Southern California**) gave a summary of **the epigenetics of cancer** (the fusion of fields, DNA methylation and chromatin structure) and a flavour of talks to come. Clusters of the unmethylated dinucleotide CpGs (more commonly known as CpG islands) are present in the promoter and exonic sequences of approximately 40% of mammalian genes, whereas other regions of mammalian genomes contain few CpG dinucleotides, which are largely methylated. $m^5C$ is mutagenic (discussed later) which probably explains the decreased occurrence of CpGs over evolutionary time. Following a comprehensive analysis of CpG islands in human chromosomes 21 and 22 [9] a new definition of CpG islands was proposed, differing from that first established by Gardiner–Garden and Frommer in 1987 [4]. The new definition includes a G + C content greater than or equal to 55% (compared to 50%) and an observed CpG over expected CpG of 0.65 (compared to 0.6) in a sequence of 500 bp or more (compared to 200 bp). Importantly, this excludes most *Alu* repetitive sequences, which are generally methylated and therefore suppressed. It is well established that the methylation of promoter CpG islands plays an important role in X chromosome inactivation, genomic imprinting, silencing of intragenic parasites and carcinogenesis.

In cancerous cells there is a general reduction of approximately 10% of the 5-methylcytosines. However there is an increase in methylation at CpG islands, including those of tumour suppressor genes (TSGs) which results in their silencing. Normal tissues also show an increase in methylation over time (aging) at CpG islands and age is the greatest risk factor in cancer. Encouragingly, hypomethylating drugs such as Zebularine may reverse the

Copyright © 2002 John Wiley & Sons, Ltd.

methylation status and therefore reactivate TSGs silenced in cancers.

Knudson's 'two-hit' hypothesis proposes that mutation of one allele and loss of the other (loss of heterozygosity) is responsible for cancer [5]. Jones concluded that one of the hits may involve methylation.

### DNA methylation and human disease

**Stephen Baylin (The John Hopkins University)** chaired and opened this session with a discussion of **promoter hypermethylation and silencing of genes in cancer**. Every major form of cancer has a contribution from promoter hypermethylation [3] but what happens at promoters that leads to hypermethylation and perhaps tumours? To address this question Baylin and colleagues studied E-cadherin CpG islands in normal and cancerous cells. They found that over time (aging) methylation boundaries are breached, resulting in inappropriate methylation of CpG islands and suppressed transcription. Suppression of TSGs gives rise to neoplasia. DNA methyltransferases (DNMTs) interact with multiple different factors including histone deacetylases (HDACs). DNMTs target unmethylated CpG islands and the subsequent methylation of these CpG islands attracts the methyl binding protein (MBP) machinery of the cell. Experiments using inhibitors of DNMTs, such as 5-aza-cytidine, show that methylation behaves as a lock for transcriptional silencing. Histone deacetylase prevents transcription and therefore there is a synergy between acetylation and methylation.

In an attempt to identify novel TSGs a microarray containing 10 000 genes was used to study gene expression in colon cancer. Approximately 250 genes were observed with altered expression and the subset of these genes containing CpG islands were all methylated in the tumours. To confirm the TSG role of one such gene (HIC1), knockout experiments were performed on heterozygous HIC1 mice. Methylation was not observed on the mutated allele when this was the only allele inherited, consistent with Knudson's 'two-hit' hypothesis [5].

**Brian Hendrich (Centre for Genome Research, University of Edinburgh)** discussed the role of **methyl-CpG binding proteins in development and disease**. Rett syndrome is a disturbing condition in which, after a period of apparently normal development, children lose the ability to speak and walk, developing incessant hand-wringing motions, seizures and scoliosis. The syndrome is the result of mutations in the ubiquitously expressed methyl-CpG binding protein MeCP2. The MeCP2 gene resides on the X chromosome and therefore, as a result of mosaic inactivation, females carrying heterozygous MeCP2 mutations deteriorate and then plateau, whilst males null for MeCP2 die within two years. Most missense mutations occur in two known functional domains and a website containing all known mutations in the MeCP2 gene can be found at: **http://homepages.ed.ac.uk/skirmis/**. MeCP2 knockouts in mice have provided a model for the subsequent study of Rett syndrome and show the requirement for MeCP2 within the central nervous system.

**Gerd Pfeifer (City of Hope National Medical Center)** continued with the theme of methyl-CpG binding domain proteins (MBDs) and in his short talk introduced **a new member of the MBD family, MBD3L**. MBD3L, a gene homologous to the methyl-CpG binding domain protein genes MBD3 and MBD2, is expressed specifically in postmeiotic cells of the testis. MBD3L has a coiled-coil domain but no MBD domain. A protein complex containing MBD2b and MBD3L results in the demethylation of the paternal genome immediately after fertilization. A transcriptional repressor domain is present at the N-terminus of MBD3L and functional assays illustrate its role as a transcriptional repressor.

Knockout experiments have shown that methylation is essential for correct cell functioning, however there is a price to pay for regulation by methylation since methylation is toxic. The deamination of cytosine gives rise to uracil but the deamination of $m^5C$ results in thymine i.e. a G/T mismatch. However MBD4 has a repair domain similar to a DNA glycosylase, which is able to remove the mismatched base.

**Ulrike Hardeland (Institute of Medical Radiobiology)** presented the **mechanisms coordinating the repair of G/T mispairs arising through methylcytosine deamination**. Human thymine-DNA glycosylase (TDG) has a 410 amino acid open reading frame encoding a protein of 46 KDa, which is highly conserved in evolution. This monofunctional DNA glycosylase has a number of implicated biological roles including; base excision repair, transcriptional regulation of gene expression and DNA methylation. A mouse with a TDG knockout shows embryonic lethality at day 10.5 post coitum.

Copyright © 2002 John Wiley & Sons, Ltd.

The interaction partners of TDG have been found using a yeast 2-hybrid assay and include the small ubiquitin-like modifiers SUMO-1 and SUMO-3 [6]. TDG is modified by sumoylation at a single lysine residue (330 of human TDG) and this SUMO-TDG shows altered substrate processing. G/U is processed by SUMO-TDG whereas G/T is not. In addition to TDG and MBD4 there are several other DNA glycosylases; uracil DNA glycosylase (UNG) and single strand selective monofunctional uracil DNA glycosylase (SMUG1), so why the apparent biochemical redundancy? This may be down to cell cycle related fluctuations. TDG is most abundant during the G1 phase of the cell cycle and is completely absent during S phase. The regulation of TDG is not at the transcriptional level but rather at the protein level, by ubiquitination prior to S phase. The differential expression of TDG and UNG during the cell cycle means there is no biochemical redundancy.

So what of the functions of the DNA methyl transferases (DNMTs) in mammalian development? **En Li** (**Massachusetts General Hospital**) discussed **the functions of Dnmt1, Dnmt3a, Dnmt3b and Dnmt3L in the mouse**. Dynamic methylation changes are observed during mouse development and indicate different roles for the methyltransferases. Dnmt1 is a housekeeping hemi-methyltransferase and inactivation of Dnmt1 did not affect global methylation in the cancer cell-line studied. In contrast, Dnmt3a and b are essential for *de novo* methylation. The Dnmt3a$^{-/-}$ mutant phenotype manifests as runted mice with abnormalities in the gastrointestinal tract e.g. functional obstruction in the cecum (proximal intestine) and bleeding. Dnmt3a is not expressed in the epithelial tissue of the gut, but in the ganglia cells and smooth muscle. Dnmt3b$^{-/-}$ mutants die at E14.5–E16.5 with anterior neural tube, ventricular septal, and liver defects as well as haemorrhage and oedema. Dnmt3a/b therefore have a broad range of developmental functions, many of which are unknown. The Massachusetts group intend to study tissue specific gene ablation and identify genes/sequences methylated by Dnmt3a and 3b during development. In contrast to 3a and 3b, Dnmt3L has no enzyme activity but does have a PHD motif in common with them. Despite its name, Dnmt3L is not a methyltransferase, but it does have a similar expression pattern to 3a/b. Dnmt3L$^{-/-}$ males are sterile with impaired spermatogenesis. Although the Dnmt3L$^{-/-}$ females are fertile, embryos from pregnant Dnmt3L$^{-/-}$ mothers die at around E10.5 and interestingly, maternally methylated imprinted genes such as Igf2r and Peg1 are hypomethylated in these embryos. Methylation of paternally imprinted genes such as H19 and Igf2 are unaffected, suggesting that Dnmt3L may cooperate with 3a/b to regulate spermatogenesis and maternal genomic imprints. Indeed, a recent paper by Bourc'his and colleagues [2] confirms that Dnmt3L is critical in the establishment of maternal genomic imprints.

### Epigenetic mechanisms in tumorigenesis

**Ina Rhee** of Bert Vogelstein's laboratory (**The John Hopkins University**) presented their work demonstrating that **DNMT1 and DNMT3b cooperate to silence genes in human cancer cells**. The role of methyltransferase enzymes has been largely studied in mouse models in the past, however Rhee and colleagues have generated knockouts of DNMT1 and DNMT3b in human colorectal cell-lines. The HCT116 colorectal carcinoma line was used because it exhibits hypermethylation-associated gene silencing, a diploid karyotype and susceptibility to targeted homologous integration. LoxP recombination was used in the targeted deletion of the DNMT1 and DNMT3b genes. Human cancer cells lacking DNMT1 retained 80% of genomic methylation (including that at *Alu* and LINE repeat elements) and associated gene silencing. Disrupting the DNMT3b gene reduced global DNA methylation by less than 3%. Next, double knockouts (DKO) of DNMT1 and DNMT3b were generated in the same cells. Instead of the anticipated decrease in methylation of approximately 23%, the researchers observed a 95% decrease in genomic DNA methylation and almost complete elimination of methyltransferase activity. In the DKO cell-lines, repeat sequences were demethylated (which is indicative of global demethylation), imprinting of the IGF2 gene was lost and there was a loss of silencing of the tumour suppressor gene p16$^{INK4a}$. The normally silenced and hypermethylated TIMP-3 gene is re-expressed in the DKO cells, with associated promoter demethylation. In one of the eight DKO lines generated (DKO 8) higher global m$^5$C was seen, as well as retention of p16$^{INK4a}$ silencing and robust growth. This DKO 8 clone suggests that enzymes other than DNMT1 and DNMT3b can be used by tumour cells to maintain methylation of critical sites in some circumstances. The DKO lines therefore represent a good resource for finding new

Copyright © 2002 John Wiley & Sons, Ltd. 

methyltransferase enzymes. Since the meeting, these findings have been published in Nature [8].

### DNA methylation and development

**David Katz** (**Princeton University**) began this session with his presentation on **epigenetic regulation of mammalian growth**. Mouse distal chromosome 7 (chr7) and the syntenic human region at 11p15 have a large cluster of imprinted genes including those responsible for Beckwith-Wiedemann Syndrome (BWS) in man. The parent specific expression of autosomal genes is achieved by the imposition of epigenetic marks (such as DNA methylation) that are established in the gametes and then maintained throughout development. It is already clear that DNA methylation is functionally responsible for gene silencing in a number of imprinted genes, including H19 at mouse distal chr7. Paradoxically, another gene in the same region, Igf2, requires the presence of DNA methylation for its expression. The presence of a chromatin boundary within the imprinting control region (ICR), upstream of H19 and downstream of Igf2, is thought to block access of enhancers to Igf2 on the unmethylated maternal chromosome, keeping it silent. On the paternal chromosome, methylation blocks the formation of the boundary and therefore the Igf2 enhancers enable its expression. The boundary requires the binding of a zinc-finger protein, CCCTC binding factor (CTCF). CTCF binds multiple times in the H19 region and plays a role in the maintenance of the maternal allele in an unmethylated state. In a CTCF mutant, enhancer binding is no longer blocked, therefore allowing expression of Igf2. The presence of CTCF in both germ-lines at the time of imprinting establishment indicates that CTCF is not responsible for establishing the maternal allele in an unmethylated state. The H19 ICR is a maternal-specific origin of replication (ORC) showing 90% maternal and only 10% paternal ORC activity. These findings are consistent with the model of ORC establishment of an unmethylated state proposed by Antequera and Bird [1].

The chair of this session, **Wolf Reik** (**Babraham Institute**) presented his group's work in elucidating the **function and mechanisms of imprinting and epigenetic reprogramming**. 50 imprinted genes have been identified in mammalian genomes to date and these genes have a major effect on foetal growth, placental development and postnatal behaviour. The genetic conflict hypothesis proposes that paternally expressed genes enhance foetal growth having been selected to recruit resources from the mother for the foetus, whilst maternal genes suppress foetal growth by inhibiting resource allocation to the foetus. The growth factor Igf2 is an important regulator of foetal growth and is highly expressed both in the placenta and foetus. The expression of this growth factor determines the size and efficiency of the placenta for nutrient transfer. In BWS, patients have a very large foetus, due to errors in this region of 11p15. In order to address the role of Igf2 in placental function, an Igf2 placental specific transcript (expressed in the labyrinth trophoblast of placenta) was studied. A knockout mouse was generated in which the placental specific promoter (P0) was removed, whilst keeping the foetal promoter intact. The result of this knockout was a placental, and late foetal, growth restriction, approximately 30% smaller than the wild type. Placental transport assays in which radioactive food is ingested by the mother prior to samples being taken from the foetus, showed that in the mutant P0 mice passive diffusion is down regulated, whilst active transport is upregulated, presumably because the smaller placenta is working harder to provide for the foetus. The coordination within the same gene is remarkable, as illustrated by the two alternative transcripts of Igf2; controlling the supply of nutrients in the placenta, and controlling the demand for nutrients in the foetus.

### DNA methylation/epigenetics

**Rudolf Jaenisch** (**Whitehead Institute for Biomedical Research**) discussed **epigenetic reprogramming in the context of mammalian cloning**. His group is interested in using nuclear cloning of vertebrates as a tool for defining the epigenetic state. The major problem encountered in nuclear cloning is genomic reprogramming, which renders the technique inefficient; most embryos die before birth with severe abnormalities. Cloned mouse pups display 'large offspring syndrome' which manifests as a huge placenta and foetus, 4 to 7 times larger than the wild type. Six known imprinted genes were tested in these mice and none were expressed correctly, indicating that abnormal imprinting is the cause of large offspring syndrome. Gene expression profiling using an Affymetrix chip was used to highlight those genes with reduced or elevated expression

Copyright © 2002 John Wiley & Sons, Ltd. 

levels in the placentas of the cloned pups. 6% of the genome was observed to be deregulated. Higher survival rates are observed when using embryonic stem (ES) cells as the nuclear donors by comparison to somatic cell donors. Somatic stem cells have similarities with ES cells and therefore those surviving clones thought to have been generated from somatic cells, may actually have been cloned from rare stem cells in the cell population.

### Gene silencing and development

**Ian Wilmut** (**Roslin Institute**) added to the discussion about cloning indicating that **the study of cloned animals, through nuclear transplantation, is revealing much about epigenetic effects**. He began by saying that while sheep, cattle, mice, goats, pigs and a cat had all been cloned from somatic cells, the same teams of researchers, with the same technology had been unable to clone rabbits, rhesus monkeys, rats and dogs. Consistent with Rudolf Jaenisch's observations, the cloning was seen as repeatable, but very inefficient, and the problems observed included increased weight, respiratory difficulty, cardiopulmonary effects, miscarriage, disorders of the immune system and reduced longevity [7]. The first gene-targeted animal at the Roslin Institute was put down after suffering from respiratory problems; the autopsy showed abnormalities in both lung and kidney tissues. Importantly, the abnormalities are not always seen in disorders arising through natural reproduction and since the phenotypes are not inherited, they must be the result of epigenetic effects [10]. Work is now underway to determine how the nucleus is epigenetically reprogrammed.

### Conclusion

We are entering a new era of functional genomics, using the reference DNA sequences for a growing number of vertebrate and invertebrate genomes to assist in the study of gene function. It is clear from this meeting that gene function cannot simply be defined from knowledge of the genomic sequence but is complicated by the epigenome. The field of epigenetics is a growing one and is full of promise, both in giving a better understanding of genome function and in using this understanding to improve healthcare.

### Acknowledgement

I'd like to thank Ian Dunham for helpful discussions and the critical reading of this manuscript.

### References

1. Antequera F, Bird A. 1999. CpG islands as genomic footprints of promoters that are associated with replication origins. *Curr Biol* **9**: 661–667.
2. Bourc'his D, Xu GL, Lin CS, Bollman B, Bestor TH. 2001. Dnmt1 and the establishment of maternal genomic imprints. *Science* **294**: 2536–2539.
3. Esteller M, Corn PG, Baylin SB, Herman JG. 2001. A gene hypermethylation profile of human cancer. *Cancer Res* **61**: 3225–3229.
4. Gardiner-Garden M, Frommer M. 1987. CpG islands in vertebrate genomes. *J Mol Biol* **196**: 261–282.
5. Knudson AG Jr. 1971. Mutation and cancer: statistical study of retinoblastoma. *Proc Natl Acad Sci U S A* **68**: 820–823.
6. Melchior F. 2000. SUMO – nonclassical ubiquitin. *Annu Rev Cell Dev Biol* **16**: 591–626.
7. Ogonuki N, Inoue K, Yamamoto Y, *et al*. 2002. Early death of mice cloned from somatic cells. *Nat Genet* **30**: 253–254.
8. Rhee I, Bachman KE, Park BH, *et al*. 2002. DNMT1 and DNMT3b cooperate to silence genes in human cancer cells. *Nature* **416**: 552–556.
9. Takai D, Jones PA. 2002. Comprehensive analysis of CpG islands in human chromosomes 21 and 22. *Proc Natl Acad Sci U S A* **99**: 3740–3745.
10. Tamashiro KL, Wakayama T, Akutsu H, *et al*. 2002. Cloned mice have an obese phenotype not transmitted to their offspring. *Nat Med* **8**: 215–216.

Copyright © 2002 John Wiley & Sons, Ltd. 
